# Distinct Distribution Patterns of Potassium Channel Sub-Units in Somato-Dendritic Compartments of Neurons of the Medial Superior Olive

**DOI:** 10.3389/fncel.2019.00038

**Published:** 2019-02-19

**Authors:** Alisha L. Nabel, Alexander R. Callan, Sarah A. Gleiss, Nikolaos Kladisios, Christian Leibold, Felix Felmy

**Affiliations:** ^1^Division of Neurobiology, Department Biology II, Ludwig-Maximilians-Universität München, Planegg-Martinsried, Germany; ^2^Graduate School for Systemic Neurosciences, Ludwig-Maximilians-Universität München, Munich, Germany; ^3^Institute of Zoology, University of Veterinary Medicine Hannover, Hanover, Germany; ^4^Computational Neuroscience, Department Biology II, Ludwig-Maximilians-Universität München, Planegg-Martinsried, Germany

**Keywords:** medial superior olive, potassium channel, potassium currents, sub-cellular localization, postsynaptic integration

## Abstract

Coincidence detector neurons of the medial superior olive (MSO) are sensitive to interaural time differences in the range of a few tens of microseconds. The biophysical basis for this remarkable acuity is a short integration time constant of the membrane, which is achieved by large low voltage-activated potassium and hyperpolarization-activated inward cation conductances. Additional temporal precision is thought to be achieved through a sub-cellular distribution of low voltage-activated potassium channel expression biased to the soma. To evaluate the contribution of potassium channels, we investigated the presence and sub-cellular distribution profile of seven potassium channel sub-units in adult MSO neurons of gerbils. We find that low- and high voltage-activated potassium channels are present with distinct sub-cellular distributions. Overall, low voltage-activated potassium channels appear to be biased to the soma while high voltage-activated potassium channels are more evenly distributed and show a clear expression at distal dendrites. Additionally, low voltage-activated potassium channel sub-units co-localize with glycinergic inputs while HCN1 channels co-localize more with high voltage-activated potassium channels. Functionally, high voltage-activated potassium currents are already active at low voltages near the resting potential. We describe a possible role of high voltage-activated potassium channels in modulating EPSPs in a computational model and contributing to setting the integration time window of coincidental inputs. Our data shows that MSO neurons express a large set of different potassium channels with distinct functional relevance.

## Introduction

Neurons in the medial superior olive (MSO) detect interaural time differences (ITDs) in the microsecond time range by an exquisitely precise integration mechanism (Grothe et al., 2010). The temporal precision of this postsynaptic integration depends on the interaction of dendritic excitation with somatic inhibition (Couchman et al., 2012; Myoga et al., 2014) under the control of voltage-activated ion channels (Scott et al., 2005, 2010; Mathews et al., 2010; Baumann et al., 2013; Roberts et al., 2013; Myoga et al., 2014).

So far only a small number of voltage-gated ion channels have been described in MSO neurons. Pharmacological and immunohistochemical evidence have shown the presence of Kv1.1 (Scott et al., 2005; Mathews et al., 2010; Roberts et al., 2013), HCNs (Khurana et al., 2011; Baumann et al., 2013), Kv3.3 (Grigg et al., 2000; Li et al., 2001) and perisomatic Nav (Scott et al., 2010). HCN1 channels set the membrane potential, contribute to the low input resistance of MSO neurons, counteract inhibitory summation (Baumann et al., 2013), and sharpen the coincidence detection window (Khurana et al., 2012). Koch et al. (2004) show that these channels are expressed in the somatic and dendritic compartments of MSO neurons. Block of Kv3 channels broadens the large action potentials in mice (Fischl et al., 2016). As action potentials in adult gerbil MSO neurons are very small, the presence and function of high voltage-activated potassium channels is unclear. Kv1.1 channels appear to be expressed with a sub-cellular gradient, decreasing toward the distal dendrites (Mathews et al., 2010). These channels generate low voltage-activated currents, whose expression profile indicates a functional relevance in reducing the coincidence detection window (Scott et al., 2005; Mathews et al., 2010) and in interacting with local inhibition to achieve microsecond precise ITD detection (Myoga et al., 2014).

The restriction of Kv1.1 channel expression to the soma and proximal dendrite indicates that other voltage-activated potassium channels might be cooperating at distal dendrites to generate the required outward currents, counteracting the presence of hyperpolarization-activated cation channels (HCN). Furthermore, dendritic excitatory postsynaptic potentials (EPSPs) are likely to reach high voltage levels (Mathews et al., 2010) suited to gate different sets of higher voltage-activated potassium channels. Therefore, we hypothesize that additional voltage-activated potassium channels are likely to be present in neurons of the mature MSO.

Below, we describe the somato-dendritic distribution of seven potassium channel sub-units in the mature MSO. Each of these compartments expresses both low and high voltage-activated potassium channels, yet with different combinations of channel sub-units. In contrast, the counter-balancing HCN1 channel is expressed evenly throughout MSO neurons. Thus, ultra-fast coincidence detection most likely relies on the interplay between synaptic input and a variety of potassium currents expressed at distinct cellular compartments.

## Materials and Methods

All animal procedures were in accordance with the guidelines of the Regierung of Oberbayern and the Deutsches Tierschutzgesetz and were approved by the local authority’s ethics committee (55.2-1-54.2531.8-211-10).

### *In vitro* Slice Preparation and Electrophysiology

Slices were prepared from Mongolian gerbils (*Meriones unguiculatus*) of either sex of postnatal day (P) 45–65. Animals were anesthetized with isoflurane and decapitated. Brains were removed in dissection solution containing (in mM) 50 sucrose, 25 NaCl, 25 NaHCO_3_, 2.5 KCl, 1.25 NaH_2_PO_4_, 3 MgCl_2_, 0.1 CaCl_2_, 25 glucose, 0.4 ascorbic acid, 3 *myo*-inositol and 2 Na-pyruvate (pH 7.4 when bubbled with 95% O_2_ and 5% CO_2_). Subsequent to brain removal, 120 μm horizontal slices were taken with a VT1200S vibratome (Leica). Slices were incubated in recording solution (same as slice solution but with 125 mM NaCl, no sucrose and 1.2 mM CaCl_2_ and 1 mM MgCl_2_ at 36°C for 15–45 min, bubbled with 5% CO_2_ and 95% O_2_).

After incubation, slices were transferred to a recording chamber attached to a microscope (BX50WI; Olympus) equipped with gradient contrast illumination and continuously perfused with recording solution. Cells were visualized and imaged with a TILL Photonics system composed of an Imago Retica DC2000 camera, a monochromator and its control unit. Whole-cell recordings were performed using an EPC10/2 amplifier (HEKA Elektronik) on visually identified MSO neurons at 34–36°C. Data were acquired at 50 kHz and low-pass filtered at 3 kHz. For voltage clamp recordings, the access resistances were compensated to a residual of 2 MΩ (access resistance ranged between 4.3 and 8.9 MΩ with the appropriate compensation ranging between 54 and 78%). Whole-cell potassium currents were pharmacologically isolated by SR95531 (SR; 10 μM), strychnine (Stry; 0.5 μM), DNQX (20 μM), DAP5 (50 μM), ZD7288 (50 μM), Cd^2+^ (100 μM), and TTX (1 μM). The intracellular solution was (in mM): 65 *K*-gluconate, 80 Na-gluconate, 4.5 KCl, 15 HEPES, 2 Mg-ATP, 2 K_2_-ATP, 0.3 Na_2_-GTP, 7.5 Na_2_-Phospocreatine, 5 Na-EGTA and 20–50 μM Alexa Fluor 568 (pH adjusted with NaOH to 7.3) leading to a calculated liquid junction potential of ∼14 mV and a calculated potassium reversal of -89 mV (Figure 6). For recordings in Figure 5 the external KCl concentration was raised to 5 mM leading to a calculated potassium reversal of -70.5 mV. Single somatic (Figure 6) and dual recordings from soma and dendrite were performed in current clamp with an internal solution containing in mM: 145 *K*-gluconate, 4.5 KCl, 15 HEPES, 2 Mg-ATP, 2 K_2_-ATP, 0.3 Na_2_-GTP, 7.5 Na_2_-phosphocreatine, 5 K-EGTA and 50 μm Alexa Fluor 488 or 568 (LJP ∼16 mV, pH adjusted with KOH to 7.25). For single recordings the electrode resistance was ∼4 MΩ; for somatic and dendritic recordings, electrode resistance in bath was 7–10 MΩ. Series resistance was below 20 MΩ in both somatic and dendritic recording sites used for analysis. For all electrophysiological data the liquid junction potential was corrected offline.

### Immunofluorescence and Confocal Microscopy

Immunofluorescence was carried out on free floating slices taken from animals of P50 to 100. Animals were anesthetized (Narcoren, Pentobarbital-Natrium, 20 mg/kg) and perfused with phosphate-buffered saline (PBS) containing 0.1% Heparin and 155 mM NaCl for about 3 min before switching the perfusion to 4% paraformaldehyde. After 25–30 min of perfusion the brains were removed and post-fixed for 3 h or overnight. Brains were washed 3 times in PBS at room temperature for 5 min each and slices of 60 μm thickness were taken with a VT1000S vibratome (Leica, Wetzlar, Germany). The slices were washed four times in PBS at room temperature for 5 min each. Consequently, they were blocked in 1 ml blocking solution (0.3% Triton, 1% Saponin, 0.1% BSA) for 1 h. After blocking, the slices were incubated in primary antibodies (AB, Table 1) diluted in 500 μl blocking solution for 48 h at 4°C on a shaker and subsequently washed 8 times in PBS at room temperature for 5 min each. Additional information regarding the primary ABs is given below. Slices were incubated in secondary ABs (Table 2) diluted in 500 μl blocking solution at room temperature for 4 h. Then the slices were mounted in Vectashield medium (H-1000, Vector Laboratories Inc., AXXORA, Lörach, Germany) and sealed with nail polish. Confocal scans were taken with a Leica TCS SPL System (Leica, Wetzlar, Germany). MSO overview images were obtained with a 63× objective (1.32 NA) leading to a pixel size of 481.47 nm ^∗^ 481.47 nm. Importantly, the images shown represent maximal intensity projections of a 7 image stack with an inter-image distance of 290 nm. Therefore, the displayed dendrites correspond to a 2 μm optical section. Since the radius of MSO dendrites ranges from 1 to 2.5 μm thickness at the investigated locations (Rautenberg et al., 2009) most of the radial extent of a dendrite is collapsed into a single image. High magnification images were generated from scanned stacks with a 63× objective with a 2× zoom, leading to a voxel size of 241.03 nm ^∗^ 241.03 nm ^∗^ 293.73 nm. Images used to extract single MSO neurons off-line were acquired with a 63× objective using 1.7× zoom, leading to a voxel size of 282 nm ^∗^ 282 nm ^∗^ 293.73 nm. Scan intensity and gain were kept constant for all images regarding a given Kv sub-unit. The intensity of gray scale Kv images and images with blocking peptide are scaled equally to compare the impact of the blocking peptide. Consecutive dual or triple wavelength line scans were always averaged 5 times. Z-chromatic shift between color channels was corrected for red–green–blue (RGB) stacks in ImageJ. Montages of RGB optical sections and maximum-intensity projections were assembled into tables by using custom-written ImageJ plugins and Adobe Photoshop CS software.

**Table 1:** Primary antibodies used in this study.

Antigen	Host	Type	Amino acid residues	Use with secondary AB conjugate with	Dilution	Company	Cat#
HCN1 N70/28	Mouse	Monoclonal	778–910	AMCA	1:500	NeuroMab	75-110
Kv1.1	Rabbit	Polyclonal	416–495	Cy3/A488	1:200	Alomone labs	APC-009
Kv1.2	Mouse	Polyclonal	428–499	DyLight 549	1:500	NeuroMab	75-008
Kv1.6	Rabbit	Polyclonal	463–530	Cy3	1:200	Alomone labs	APC-003
Kv2.1	Rabbit	Polyclonal	841–857	Cy3	1:200, 1:1000	Alomone labs	APC-012
Kv2.2	Rabbit	Polyclonal	859–873	Cy3	1:200	Alomone labs	APC-120
Kv3.1b	Rabbit	Polyclonal	567–585	Cy3	1:200	Alomone labs	APC-014
Kv3.2	Rabbit	Polyclonal	184–204	Cy3	1:200	Alomone labs	APC-011
MAP2	Chicken	Polyclonal		AMCA/A488	1:1000	Neuromics ACRIS	CH22103
S100β	Rabbit	Polyclonal		A488	1:1000	Swant	37
SV2	Mouse	Monoclonal		A488	1:500	DSHB	SV-a1 SV2c
GlyT2	Rabbit	Polyclonal	1–229	Cy3	1:1000	Synaptic Systems	272003


**Table 2:** Secondary antibodies used in this study.

Antigen	Conjugate	Host	Dilution	Company	Cat#
Anti-mouse	AMCA	Donkey	1:100	Dianova	
Anti-mouse	Alexa488	Donkey	1:200	Invitrogen	A21202
Anti-mouse	DyLight 549	Goat	1:500	Dianova	115-505-207
Anti-rabbit	Cy3	Donkey	1:400	Dianova	711-165-152
Anti-rabbit Fab	Alexa488	Donkey	1:100	Dianova	711-547-003
Anti-chicken	AMCA	Donkey	1:200	Dianova	703-156-155
Anti-chicken	Alexa488	Donkey	1:300	Dianova	703-546-155


Antibody information: The Kv1.1 antibody used here was verified by Zhou et al. (1998) in knock-out mice. We used the same dilution as these authors. The Kv1.2 antibody is knock-out verified by the company NeuroMab. The specificity of this antibody in gerbils can be derived from its precise labeling in the hemi-nodes of MSO axons (Lehnert et al., 2014). Kv2.2 was knock-out verified by Johnston et al. (2008) and Tong et al. (2013). In these studies the presence of Kv2.2 was shown in medial nucleus of the trapezoid body (MNTB) neurons of mice. Kv3.1b and Kv3.2 were also knock-out verified by Kudo et al. (2011) and Barry et al. (2013), respectively. The antibody used to detect Kv1.6 was illustrated to selectively label cells that were also positive for Kv1.6 mRNA (Smart et al., 1997), indicating high specificity. For the antibody directed against Kv2.1 no knock-out verification has been demonstrated so far. To further test the antibody’s specificity we included an additional control with a low dilution and also tested the expression in cells that are known to be Kv2.1 positive. Despite our effort to use validated antibodies, standard staining procedures and blocking peptides, we are aware of the limitations and cannot fully rule out off-target protein detection in the Mongolian gerbil.

### Data Analysis

Data were analyzed using Igor Pro (Wavemetrics), ImageJ and Excel. The intensity distribution profiles for Kvs, HCN1, glycine transporter 2 (GlyT2) and microtubule associated protein 2 (MAP2) were extracted by performing line scans (line width: 100 pixels) orthogonally to the MSO dorso-ventral axis on low magnification, maximum intensity projection images using ImageJ. Line scans were taken in the central third of the MSO dorso-ventral extent. These line scans were then fitted with a Gaussian function (IgorPro) and their half-widths were collected. Since the neurons in the MSO are mainly bipolar shaped (Rautenberg et al., 2009) and aligned into a columnar arrangement in gerbils, a Gaussian fit most adequately captures the profile of the fluorescence distribution. The average ratios of Kvs, HCN1 or GlyT2 to MAP2 half-widths were then calculated. A value close to one indicates a distribution largely biased to the somatic/perisomatic region, since MAP2 shows highest expression at the soma and proximal dendrites. Values larger than one indicate a broad, dendritic distribution profile.

For the single cell analysis, MSO neurons were manually labeled using the paintbrush tool in ImageJ to carefully follow the cell through each optical section of the confocal stack. Subsequently, the region outside the paintbrush label was deleted (digital extraction). Then a line scan (width: 9 pixels) was performed along the longitudinal axis of each of these neurons. The average intensity of the first ten pixels from the edge of the cell’s nucleus was used for normalization. Only dendrites of at least 75 μm length were taken into account. This dendritic length covers the previous immunofluorescent data in Couchman et al. (2010), and the distance of dendritic recordings (Mathews et al., 2010; Winters et al., 2017) and approximates about two thirds of the overall average length (Rautenberg et al., 2009). Results are presented as mean ± SEM.

### Compartmental Modeling

We simulated a minimal multi-compartmental model where a somatic compartment of 30 μm length was sandwiched between two dendrites, each having a total length of 150 μm and consisting of 10 compartments (Figure 7B). Each compartment was cylindrical, with somatic diameter 15 μm and tapered dendrites such that the most proximal dendritic compartments had diameter 4.4 μm and the most distal 1.7 μm. The specific axial resistance connecting the compartments was taken as 200 Ωcm, and the specific membrane capacitance as *C* = 0.9 μF/cm^2^ (Gentet et al., 2000). Geometry was adjusted such that the total cell capacitance was approximately 40 pF as previously reported (Rautenberg et al., 2009).

The membrane potential of each compartment was modeled according to a Hodgkin–Huxley type equation:

$$C_{m}\frac{dV}{dt}=-\Sigma I_{ionic}$$

Where *I*_ionic_ includes axial current between compartments (Lehnert et al., 2014) and transmembrane currents.

In addition to a leak current with 0.05 mS/cm^2^ conductance, all compartments were equipped with three active transmembrane currents: (1) A low-threshold potassium current (KLT) modeled according to Mathews et al. (2010). (2) An HCN current modeled according to Baumann et al. (2013) using the parameters for dorsal MSO neurons. (3) A high-threshold potassium current (KHT) modeled based on parameters measured in this study (Figure 6). This modeled current is supposed to comprise all molecular sub-types of high voltage-activated potassium channels that we have detected by immunofluorescence.

KHT was modeled as

$$I(V)=g_{KHT,peak}X^{2}\left(V-E_{K}\right)$$

where the gating variable *X*, followed the equation

$$\frac{dX}{dt}=\frac{X_{\infty }-X}{\tau_{X}}$$

with

$$X_{\infty }(V)=\frac{1}{1+e^{\frac{-\left(V+44.9\right)}{30}}}$$

(voltage in mV). The square of the activation function was fitted to the measured peak activation function (filled circles in Figure 6B), thereby accounting for the exponent 2 in the conductance term. As time constants, we used τ_x_ = 0.8 ms or 1.5 ms as specified in the results section.

Three variants of the model were tested, with peak channel conductance as outlined in Table 3.

**Table 3:** Peak conductances, in mS/cm^2^, for each variant of the model.

Model variant	KLT	KHT	HCN
KLT only	63.4	0	1.265
KHT only	0	1.152	2.58
KLT and KHT	29.9882	0.5449	1.91


The peak channel conductance of KLT was implemented as distance dependent according to

$$g_{KLT}(X)=g_{KLT,peak}\left(1+1.5e^{\frac{-X}{22}}\right)$$

while the conductance was equal in all compartments for HCN and KHT.

The conductance amplitudes were chosen to fit the physiological resting potential and the input resistance recorded at the soma. The reversal potential of potassium was taken as -90 mV, of the HCN current as -35 mV, and of the leak current as -70 mV.

## Results

The expression profiles of seven voltage-activated potassium channel sub-units were investigated in neurons of the MSO in adult Mongolian gerbils. The classification of sub-units that contribute to the different types of potassium currents was based on Gutman et al. (2005). To quantify the sub-units’ sub-cellular distribution, sections were co-stained with the cellular marker MAP2. The MAP2 staining was used to delineate the somato-dendritic extent of MSO neurons and to evaluate distribution patterns.

### Low Voltage-Activated Potassium Channels

**FIGURE 1:**
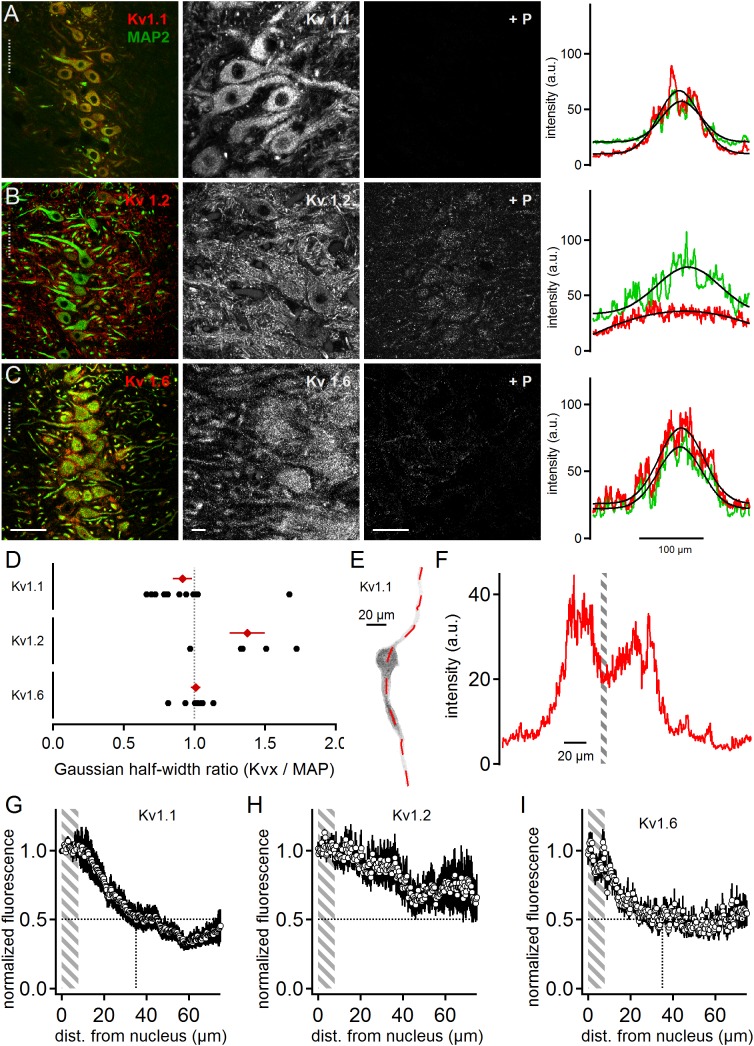
Low voltage-activated potassium channels in medial superior olive (MSO) neurons. **(A)** Immunofluorescent staining of Kv1.1, MAP2 (co-staining left, magnified Kv1.1 image middle) and Kv1.1 blocking peptide (+P, right). Intensity scaling of the gray scaled images is identical, to indicate the effect of the blocking peptide. Line scan intensity profiles are shown as arbitrary units (a.u.) on the right. The line scan was taken from the left image at the position of the gray dotted line. Colors match the color code in the left image. The black line indicates a Gaussian fit on the intensity distributions. Scale bars: left 50 μm, middle 10 μm, right 50 μm. **(B)** Same as in **(A)** but for Kv1.2 sub-unit staining. **(C)** Same as in **(A)** but for Kv1.6 sub-unit staining. **(D)** Quantification of the intensity distributions shown in **(A–C)**. The half width of the Gaussian fit was used to calculate the potassium channel to MAP2 ratio for Kv1.1 (*n* = 14), Kv1.2 (*n* = 5), and Kv1.6 (*n* = 8). Black symbols represent single images, red symbols represent average values. The gray dotted line indicates distribution profile equivalent to that of MAP2. Large values indicate a broader, more dendritic distribution profile. **(E)** Single, digitally extracted MSO neuron stained for Kv1.1. The position of the line scan is given by the red dotted line. **(F)** Intensity profile of the line scan shown in **(E)**. Gray area indicates the region of the cell’s nucleus. **(G)** Normalized intensity distribution of Kv1.1 (*n* = 19) in single MSO neurons. The edge of the nucleus was defined as zero position. Gray area indicates the region of the soma. Dotted horizontal line indicates the half decay of the normalized intensity. Dotted vertical line indicates the position the intensity reached half of its initial value. **(H)** Same as in **(G)** but for Kv1.2 (*n* = 7) sub-unit staining. **(I)** Same as in **(G)** but for Kv1.6 (*n* = 10) sub-unit staining.

All three low voltage-activated potassium channel sub-units tested were present in MSO neurons (Figure 1). Kv1.1 expression appeared biased to the somatic region of MSO neurons and restricted to the postsynaptic membrane surface (Figure 1A). The quality and specificity of the Kv1.1 staining was verified by the application of the specific control blocking peptide (Figure 1A). In a first gross analysis the expression patterns of MAP2 and Kv1.1 were compared. Toward this aim, line scans orthogonal to the dorso-ventral axis of the MSO were performed on co-stained images. The spatial intensity distribution (arbitrary units, a.u.) of the Kv1.1 and MAP2 line scans was fitted with a Gaussian function to obtain the distribution half-width (Figure 1A). From these half-widths the Kv-to-MAP2 ratio was calculated. The average Kv1.1 to MAP2 ratio was 0.92 ± 0.07 (*n* = 14, Figure 1D). A ratio of one indicated that expression profiles of MAP2 and Kv1.1 were similar. Since MAP2 expression was biased to the soma and proximal dendrites of neurons, this therefore indicated that Kv1.1 expression was more prominent at the soma, confirming previous electrophysiological evidence (Mathews et al., 2010).

Kv1.2 staining was more broadly distributed (Figure 1B) compared to Kv1.1. The higher magnification image indicates somatic and dendritic expression in the postsynaptic membrane domain, and also a possible contribution of presynaptic elements (Figure 1B). The blocking peptide strongly suppressed the Kv1.2 antibody staining, although a weak background of unspecific staining remained (Figure 1B). Irrespective of a possible presynaptic bias, line scans were performed from the co-stained image and their intensity distributions fit with a Gaussian (Figure 1B). Calculating the Kv1.2 to MAP2 ratio yielded an average value of 1.37 ± 0.12 (*n* = 5, Figure 1D). This indicated that Kv1.2 showed relatively higher dendritic expression with respect to the soma when compared to Kv1.1 sub-units.

Kv1.6 is another DTX-sensitive, low voltage-activated potassium channel (Gutman et al., 2005). Kv1.6 staining was more prominent at the soma but also indicated some expression at the dendrite (Figure 1C). The specificity of this staining pattern was supported by the nearly complete loss of fluorescence induced by the blocking peptide (Figure 1C). The somatically biased staining was quantitatively verified by the line scan analysis (Figure 1C,D) yielding an average ratio of 1.01 ± 0.03 (*n* = 8; Figure 1D). Therefore, Kv1.6 and Kv1.1 appeared locally co-expressed toward the soma, while Kv1.2 covered a larger part of the dendritic extent.

So far, our line scan analysis was based on images of the entire MSO and took all cellular components within this nucleus into account. To restrict the expression analysis to the postsynaptic somato-dendritic compartment, single MSO neurons were digitally extracted from the image stacks [(Couchman et al., 2010); Figure 1E]. This allowed a line scan analysis exclusively of the somato-dendritic compartment (Figure 1E,F), from which the region of the nucleus of an MSO neuron could be determined (Figure 1F). The borders of the nucleus were then used to align the spatial extent of line-scans from different dendrites. By this alignment the somatic region covered only about 8 μm (Figure 1G–I). The normalized Kv1.1 (dendrites: *n* = 19, Figure 1G) and Kv1.6 (*n* = 10, Figure 1I) fluorescence decayed rapidly from the soma to 50% at a dendritic distance of 35 and 24 μm, respectively. Kv1.2 (*n* = 7, Figure 1H) expression, however, remained above 50% of the somatic intensity for the entire dendritic distance measured here. Thus, different low voltage-activated potassium channels were expressed with distinct spatial profiles in MSO neurons, and were present in both the soma and the dendrite.

### Distribution of Kv1.1 With HCN1 and GlyT2

**FIGURE 2:**
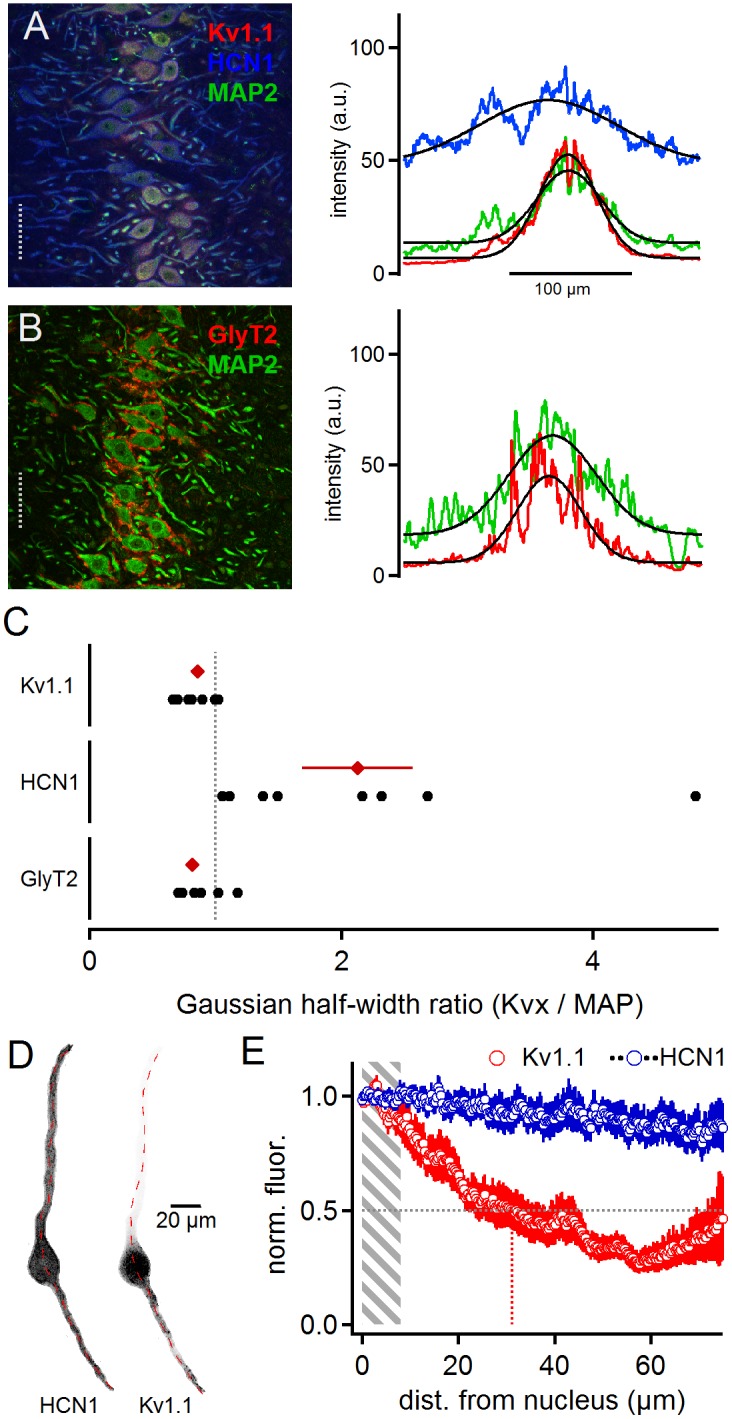
Matching distributions of Kv1.1 and GlyT2 but not HCN1. Panel **(A)** shows HCN1, KV1.1, and MAP2 co-staining (left). The lines scan intensity profiles are shown as arbitrary units (a.u.) on the right. The line scan was taken from the left image at the position of the gray dotted line. Colors match the color code of the left image. The black line indicates a Gaussian fit on the intensity distributions. Scale bar equals 50 μm. Panel **(B)** shows GlyT2 and MAP2 co-staining. **(C)** Quantification of the intensity distributions shown in **(A,B)**. The half width of the Gaussian fit was used to calculate the GlyT2 (*n* = 11), HCN1/KV1.1 (*n* = 8) to MAP2 ratio. Black symbols represent single images, red symbols represent average values. The gray dotted line indicates a distribution profile equivalent to that of MAP2. Larger values indicate a broader, more dendritic distribution profile. **(D)** Single, digitally extracted, MSO neuron co-stained for HCN1 and Kv1.1. The position of the line scan is given by the red dotted line. **(E)** Normalized intensity distribution of HCN1 and the co-stained Kv1.1 (*n* = 12) in single MSO neurons. The edge of the nucleus was defined as zero position. Gray area indicates the region of the soma. Gray dotted line indicates the half decay of the normalized intensity. Colored dotted line indicates the position the intensity reached half of its initial value.

Low voltage-activated currents have been proposed to interact with *I*_h_ currents (Khurana et al., 2011) mediated by HCN1 and HCN2 sub-units (Koch et al., 2004; Khurana et al., 2012; Baumann et al., 2013). To detect overlapping distribution profiles where these channels can directly interact for compartmentalized voltage signaling, we stained cells for Kv1.1, HCN1 and MAP2 (Figure 2B). The HCN1 sub-unit was broadly distributed (Figure 2A). The line scan analysis of these triple labeled samples (Figure 2A) leads to an average HCN1 to MAP2 ratio of 2.13 ± 0.44 and Kv1.1 to MAP2 ratio of 0.86 ± 0.05 (*n* = 8, Figure 2C). Moreover, low voltage-activated potassium currents have been suggested to interact with the voltage signaling of glycinergic inhibition (Myoga et al., 2014). Therefore, we also co-stained for GlyT2 and MAP2 proteins (Figure 2B). For GlyT2 and MAP2 the gross line scan analysis revealed a ratio of 0.82 ± 0.05 (*n* = 11, Figure 2C). Therefore, as demonstrated before (Clark, 1969; Kapfer et al., 2002; Couchman et al., 2010, 2012), inhibitory inputs are constrained to the soma and proximal dendrite (Figure 2A), and thus they localize exquisitely well with Kv1.1, indicative of a functional interaction between glycinergic inhibition and Kv1.1 activation (Myoga et al., 2014), whereas HCN channels were evenly distributed and only partially overlapped with Kv1.1. The dichotomy between Kv1.1 and HCN1 distribution was verified by single cell analysis (Figure 2D,E). On average, the normalized Kv1.1 fluorescence reached half maximum at 31.1 μm distance from the nucleus, whereas HCN1 fluorescence remained stable over the entire dendritic extent (*n* = 12). Thus, the differential distribution of HCN1 and Kv1.1 indicates that their proposed interplay (Khurana et al., 2011) is limited to the somatic region and that at distal dendrites HCN1 contributes to voltage signaling decoupled form Kv1.1.

### High Voltage-Activated Potassium Channels

Kv2.x and Kv3.x activate at higher voltage levels compared to Kv1.x channels (Gutman et al., 2005; Johnston et al., 2008). In MSO neurons, dendritic depolarizations might reach such voltage levels (Mathews et al., 2010) and therefore, potentially activate high voltage-activated potassium channels. We therefore stained for four high voltage-activated potassium channel sub-units to determine whether they could potentially contribute to voltage signaling in MSO neurons.

**FIGURE 3:**
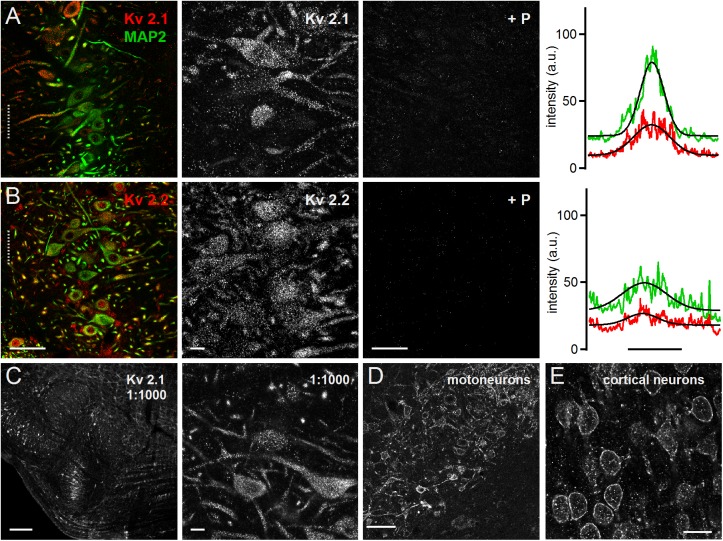
Medial superior olive neurons express Kv2.1 and Kv2.2. **(A)** Immunofluorescent staining of Kv2.1, MAP2 (co-staining left, magnified Kv2.1 image middle) and Kv2.1 blocking peptide (+P, right). Intensity scaling of the gray scaled images is identical, to indicate the effect of the blocking peptide. Line scan intensity profiles are shown as arbitrary units (a.u.) on the right. The line scan was taken from the left image at the position of the gray dotted line. Colors match the color code in the left image. The black line indicates a Gaussian fit on the intensity distributions. Scale bars: left 50 μm, middle 10 μm, right 50 μm. **(B)** Same as in **(A)** but for Kv2.2 sub-unit staining. **(C)** Control staining of higher Kv2.1 antibody dilution of the superior olivary complex (left) and in the MSO (right). Scale bars equal 200 μm (left) and 10 μm (right). **(D,E)** Control images of brain regions of well documented Kv2.1 expression. **(D)** Moto-neurons in the brainstem of the facial nerve. Standard antibody dilution of 1:200 was used. **(E)** Pyramidal neurons in cortex. Scale bars equal 100 μm.

**FIGURE 4:**
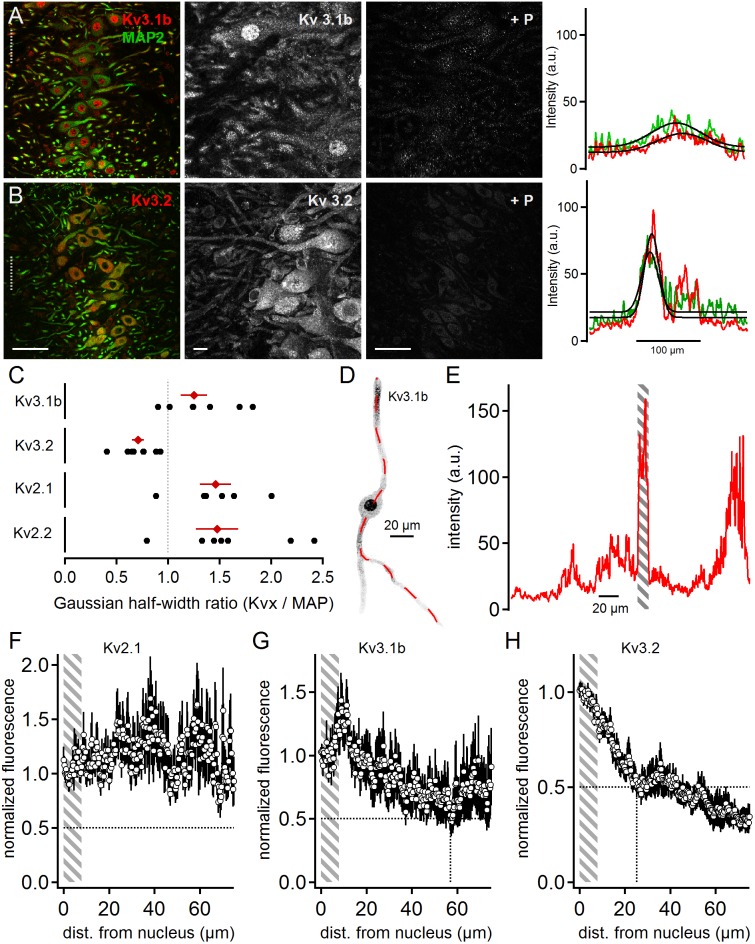
High voltage-activated potassium channels in MSO neurons. **(A)** Immunofluorescent staining of Kv3.1b, MAP2 (co-staining left, magnified Kv3.1b image middle) and Kv3.1b blocking peptide (+P, right). Intensity scaling of the gray scaled images is identical, to indicate the effect of the blocking peptide. Line scan intensity profiles are shown as arbitrary units (a.u.) on the right. The line scan was taken from the left image at the position of the gray dotted line. Colors match the color code in the left image. The black line indicates a Gaussian fit on the intensity distributions. Scale bars: left 50 μm, middle 10 μm, right 50 μm. **(B)** Same as in **(A)** but for Kv3.2 sub-unit staining. **(C)** Quantification of the intensity distributions shown in **(A,B)** and for Kv2.1 and Kv2.2 staining shown in Figure 2. The half width of the Gaussian fit was used to calculate the potassium channel to MAP2 profile for Kv3.1b (*n* = 7), Kv3.2 (*n* = 8), Kv2.1 (*n* = 6), and Kv2.2 (*n* = 7). Black symbols represent single images, red symbols represent average values. The gray dotted line indicates a distribution profile equivalent to that of MAP2. Larger values indicate a broader, more dendritic distribution profile. **(D)** Single, digitally extracted MSO neuron stained for Kv3.1b. The position of the line scan is given by the red dotted line. **(E)** Intensity profile of the line scan shown in **(D)**. Gray area indicates the region of the cell’s nucleus. **(F)** Normalized intensity distribution of Kv2.1 (*n* = 13) in single MSO neurons. The edge of the nucleus was defined as zero position. Gray area indicates the region of the soma. Dotted horizontal line indicates the half decay of the normalized intensity. **(G)** Same as in **(F)** but for Kv3.1b (*n* = 6) sub-unit staining. Dotted vertical line indicates the position the intensity reached half of its initial value. **(H)** Same as in **(F)** but for Kv3.2 (*n* = 9) sub-unit staining. Dotted vertical line indicates the position the intensity reached half of its initial value.

Both Kv2.1 and Kv2.2 could be detected all along the MSO neurons from the soma to the distal dendrite (Figure 3A,B). This expression pattern was apparent in the overview and the high magnification images. For both antibodies the blocking peptide prevented all staining (Figure 3A,B), indicating high specificity. The line scan analysis for the presented overview images indicated a broader intensity profile compared to the MAP2 staining (Figure 3A,B). The average Kv2.1 and Kv2.2 to MAP2 ratio was 1.46 ± 0.15 (*n* = 6) and 1.48 ± 0.20 (*n* = 7), respectively (Figure 4C). Since Kv2.x expression is rather unusual in auditory brainstem neurons and found to be restricted to medial (MNTB) and ventral nucleus of the trapezoid body (VNTB) neurons in mouse (Johnston et al., 2008; Tong et al., 2013) we used additional Kv2.1 staining to confirm antibody specificity. First, the same staining pattern was observed for a fivefold lower antibody concentration (Figure 3C). Second, the antibody detected Kv2.1 in moto-neurons, cortical neurons (Figure 3D,E) and the cerebellum, all of which are known to express these channel types (Escoubas et al., 2002; Guan et al., 2007; Zhuang et al., 2012; Bishop et al., 2015, 2018). Particularly for cortical pyramidal neurons, the patched staining obtained here in gerbils (Figure 3E) matches the staining pattern in mice (Bishop et al., 2015, 2018). Thus, we conclude that the high voltage-activated potassium channels Kv2.1 and 2.2 are distributed rather uniformly in MSO neurons.

Positive immunostainings were also obtained for the high voltage-activated potassium channel sub-units Kv3.1b and Kv3.2 (Figure 4). This contrasts earlier reports (Li et al., 2001) that have detected Kv3.3 but not Kv3.1b expression in neurons of the rat MSO. Kv3.1b sub-units were detected in overview and magnified images in the soma and dendrites in addition to strong nuclear labeling (Figure 4A). All labeling appeared specific as indicated by the fluorescence loss by the addition of the blocking peptide (Figure 4A). Despite the strong nuclear labeling, the line scan analysis indicated a broad distribution of Kv3.1b throughout the MSO (Figure 4A). The average line scans led to a Kv3.1b to MAP2 ratio of 1.25 ± 0.13 (*n* = 7, Figure 4C). This Kv3.1b expression profile contrasted with the expression of Kv3.2, which appeared more localized to the soma (Figure 4B). In the higher magnification images weaker dendritic staining was also apparent (Figure 4B). The gross quantification by the line scans of the overview images (Figure 4B) yields a Kv3.2 to MAP ratio of 0.71 ± 0.06 (*n* = 8, Figure 4C). Thus, our data showed that high voltage-gated potassium channels were present in MSO neurons with distinct expression patterns (Figure 4C).

Line scan analysis of digitally extracted single cells was carried out for the high voltage-gated sub-units Kv2.1, Kv3.1b, and Kv3.2 (Figure 4D,E). The images and the line scans were again used to detect the edges of the nucleus (Figure 4E). The normalized and spatially aligned fluorescence values corroborated the somatic and dendritic expression patterns of Kv2.1 and Kv3.1b (Figure 4F,G). In contrast, Kv3.2 expression dropped below 50% of somatic expression at a distance of only 25 μm from the nucleus rim (Figure 4H).

### Pharmacological Verification of Expression of Multiple Potassium Channels

**FIGURE 5:**
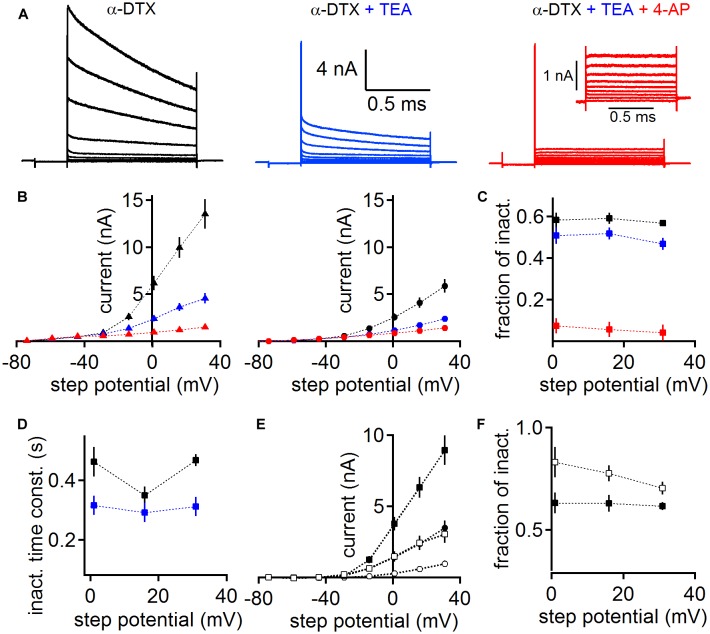
Pharmacological verification of different classes of voltage-activated potassium channels. **(A)** MSO whole-cell currents in the presence of 100 nM α-DTX (left), after the addition of 1 mM TEA (middle) and 2 mM 4-AP (right). Inset (right) shows the magnified remaining potassium current in the presence of α-DTX, TEA, and 4-AP. **(B)** The potassium peak (left) and steady state (right) current as a function of step potential. Colors represent the different pharmacological conditions: black: α-DTX; blue: α-DTX + TEA; red: α-DTX + TEA + 4-AP. The steady state current was determined at the end of the step potential. **(C)** Fraction of inactivation (peak – steady state/peak) for the potassium currents under the pharmacological conditions described in **(B)**. **(D)** Weighted decay time course of inactivation of the currents recorded under α-DTX (black) or α-DTX + TEA (blue). **(E)** The peak (square symbols) and steady state (round symbols) current of the TEA (black symbols) and 4-AP (open symbols) sensitive currents gained after subtraction. **(F)** The fractional inactivation of the TEA and 4-AP sensitive current. Symbols as in **(D)**.

Diverse potassium channels could be pharmacologically identified during voltage clamp recordings. We used subsequent application of low concentrations of tetraethylammonium (TEA 1 mM) and 4-aminopyridine (4-AP 2 mM, Figure 5) on a background of dendrotoxin (DTX) to directly demonstrate the presence of Kv3 and Kv4 type potassium channels, leaving Kv2 channels as a predominant remaining current (Gutman et al., 2005; Johnston et al., 2010). As the presence of a large low voltage-activated potassium current has been demonstrated before using DTX (Scott et al., 2005; Mathews et al., 2010; Roberts et al., 2013), we conducted this pharmacological assay on a background of 100 nM α-DTX, which blocks Kv1.1, Kv1.2, and Kv1.6.

Despite the presence of α-DTX, MSO neurons (*n* = 6) generated a large inactivating outward current (Figure 5A). This current shows peak and steady state values of about 6.17 ± 0.77 nA and 2.57 ± 0.38 nA, respectively, at 1 mV holding potential (Figure 5B). The resulting current shows a large fraction of inactivation (Figure 5C). Additional bath application of 1 mM TEA reduced the overall peak and steady state MSO potassium current (Figure 5B), leaving a remaining inactivating potassium current (Figure 5B,C). To corroborate that under the α-DTX and α-DTX/TEA conditions different currents types remain, we analyzed their inactivating time course. The weighted decay time constant was faster after the addition of TEA (Figure 5D). The subtraction of the TEA sensitive current itself is consistent with the presence of a Kv3.x driven potassium current (Gutman et al., 2005). In agreement with the presence of Kv3.x channels in auditory brainstem (Wang et al., 1998; Li et al., 2001; Chang et al., 2007; Kulesza, 2014) this TEA sensitive current had average peak and steady state values of 3.76 ± 0.48 nA and 1.42 ± 0.29 nA at a holding potential of 1 mV, respectively (Figure 5E). Thus, this TEA sensitive current showed a substantial fractional inactivation (Figure 5E). The subsequent application of 4-AP reduced the potassium current further, indicating the presence of inactivating A-type potassium current based on Kv1.4 and/or Kv4.x channels (Gutman et al., 2005; Johnston et al., 2010). The 4-AP sensitive current had an average peak amplitude of 1.47 ± 0.36 nA and a steady state amplitude of 0.28 ± 0.11 nA at a holding potential of 1 mV, and inactivated by about 50% (Figure 5E). The final remaining potassium current had an average peak amplitude of 0.93 ± 0.11 nA and a steady state amplitude of 0.86 ± 0.1 nA for a holding potential of 1 mV (Figure 5B), and hence showed little inactivation (Figure 5C). Therefore its inactivation time course was not analyzed. The pharmacological profile and the low fractional inactivation suggested that the remaining potassium current was driven by Kv2.x channels (Gutman et al., 2005; Johnston et al., 2010). Taken together, we pharmacologically verified the presence of the low and high voltage-activated potassium currents, which we have identified with immunofluorescence. In addition, our pharmacological analysis suggests the presence of further A-type potassium channel sub-units that underlie 4-AP sensitivity.

So far our data show that mature MSO neurons express many low and high voltage-activated and possibly A-type potassium channel sub-units. On the sub-cellular level each sub-unit showed expression along the full length of the cell, albeit with a somatic or dendritic bias. Each potassium current type is therefore likely to be present at each cellular compartment, yet with low voltage-activated potassium currents localized more somatically and high voltage-activated potassium currents located more dendritically. Next, we aimed to obtain functional insights into the presence of high voltage-activated potassium currents.

**FIGURE 6:**
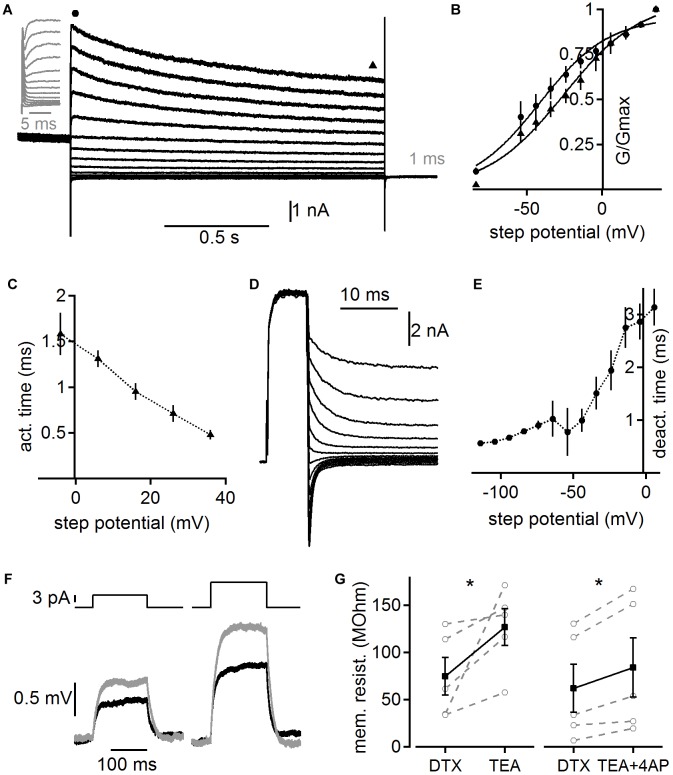
Voltage dependent kinetics of high voltage-activated potassium currents. **(A)** Potassium currents evoked by step potentials between 36 and –84 mV after a 1 s long pre-depolarization to 0 mV to pre-inactivate possible remaining low voltage-activated and A-type potassium currents. Currents recorded in the presence of 100 nM DTX. Gray inset magnifies the onset of the current. Circle and triangle highlight regions of the onset and steady state current used for further analysis shown in **(B)**. **(B)** Voltage dependent activation of potassium currents as shown in **(A)**. Circle and triangle correspond to the onset and steady state current, respectively. **(C)** Activation time constants derived from exponential fits to the onset of the potassium currents as shown in **(A)**. **(D)** High voltage-activated potassium tail currents recorded in the presence of 100 nM DTX. A 7 ms long depolarization to 36 mV was followed with step potentials between 6 and –114 mV. **(E)** Deactivation time was derived from the rapid component of a bi-exponential fit to the deactivation currents as shown in **(D)**. **(F)** Membrane deflections in response to a 5 and 10 pA current injection. Black trace depicts control recorded in the presence of DTX (100 nM), ZD7288 (50 μM), and TTX (1 μM). Gray trace shows the response following under the addition of TEA (1 mM). **(G)** Membrane resistance after additional application of TEA (1 mM) and TEA plus 4-AP (2 mM) derived from experiments illustrated in **(F)**. Asterisk indicates significance (paired *T*-Test, *p* < 0.05).

In order to obtain functional insights from a computational model we first quantified the biophysical characteristics of the overall whole-cell high voltage-activated potassium current. Toward this aim we recorded potassium currents in the presence of DTX and pre-depolarized (to 0 mV for 1 s) the cells to inactivate possible A-type currents before using step commands between -84 and 36 mV to activate high voltage-activated potassium currents (Figure 6). The voltage dependent activation of the peak and steady state conductance indicates that high voltage-activated potassium currents are already activated at low voltages in adult MSO neurons (*n* = 6). Fitting the voltage dependent activation indicates rather shallow voltage dependence, which is in accord with the presence of several channel sub-types with distinct but overlapping kinetics. The activation time of this lumped whole-cell high voltage-activated potassium current was determined by exponential fits to the rising phase of the current following the artifact. Step potentials to above -10 mV yielded fits appropriate for further analysis. The average activation time constant was voltage dependent and was below 1.5 ms above 0 mV (Figure 6C). To determine the deactivation time constant a depolarization for 7 ms to 36 mV was followed by a step command between 6 and -114 mV (Figure 6D). The resulting tail current was best fit by a double exponential function, of which the fast time constant was attributed to the high voltage-activated potassium channel’s deactivation. This analysis showed that voltage dependent deactivation ranged from 3.14 ± 0.348 ms to 0.56 ± 0.003 ms (Figure 6E). This time course is in the range with fits obtained from the tail currents to the holding command potential of -74 mV (0.532 ± 0.47 ms) of the stimulation paradigm shown in Figure 6A. Thus, our data indicate a broad voltage activation range of fairly rapidly activating high voltage-activated potassium currents with low activation thresholds that allow some of these channels to be open at the resting potential of adult MSO neurons. The broad voltage dependent activation range is likely due to the overlap of several different high voltage-activated potassium channel sub-types, consistent with our immunofluorescence.

To verify the low voltage activation of the non-DTX sensitive high voltage-activated potassium channel, we determined its impact on the membrane resistance close to resting potentials. A step current between 5 and 50 pA was applied 30 times and the average voltage deflection (Figure 6F) was used to determine the membrane resistance from the steady state level according to Ohm’s law. Cells were kept at similar potentials before (-55.8 ± 1.68 mV) and after (-57.0 ± 1.04 mV) the application of high voltage-activated potassium blockers by current injections. After DTX, TTX, and ZD7288 were present for ∼10 min in the bath TEA (1 mM) or TEA plus 4-AP (2 mM) were additionally applied. The input resistance significantly increased for both conditions from an average of 74.6 ± 20.1 to 126.5 ± 19.8 (*n* = 5; *p* = 0.04; Figure 6G) and from 62.0 ± 25.4 to 83.8 ± 31.4 MΩ (*n* = 5; p = 0.013; Figure 6G) for TEA alone and TEA plus 4-AP, respectively. Thus, high voltage-activated potassium channels contribute significantly to the resting input resistance of adult MSO neurons.

### Modeling the Impact of High Voltage-Activated Potassium Currents on Postsynaptic Integration

**FIGURE 7:**
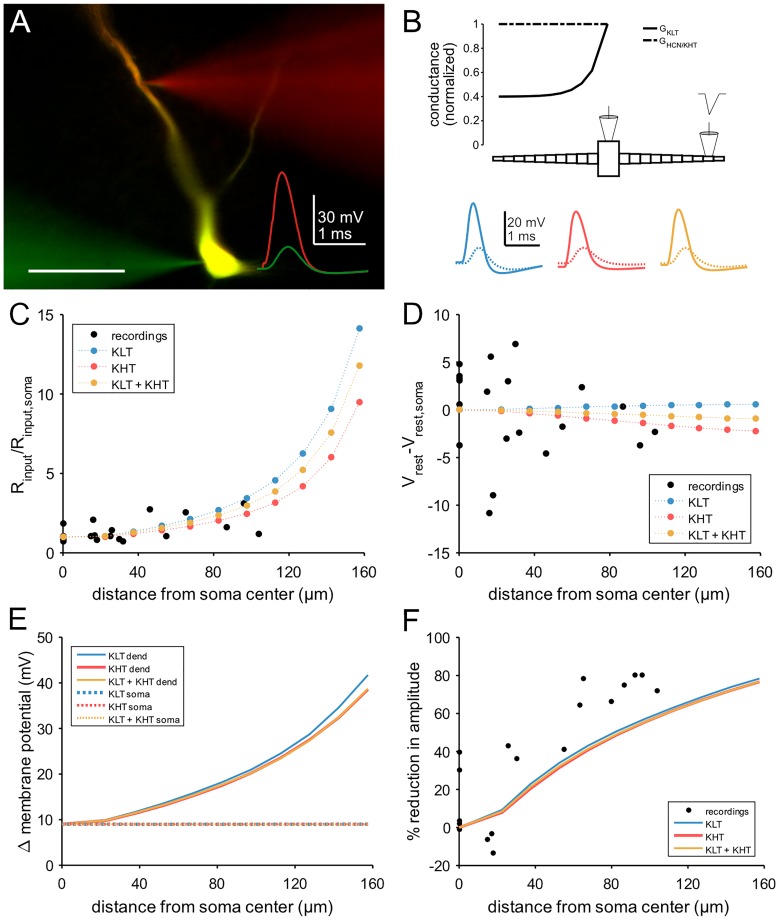
Compartmental model of an MSO neuron incorporating KHT. **(A)** Dual patch clamp in MSO. Traces show response to a triangular current pulse injected at the somatic electrode. Scale bar = 50 μm. **(B)** Schematic of compartmental model, showing spatial distribution of active conductances. Below: membrane potential of each model variant in response to the same triangular current pulse used for dual recordings, injected into a single distal dendritic compartment. Blue = KLT, red = KHT, and yellow = KLT + KHT. Solid line = dendritic compartment, dotted line = somatic compartment. **(C)** Input resistance divided by the somatic value with respect to distance from soma. **(D)** Resting membrane potential less the somatic value with respect to distance from soma. **(E)** Peak membrane voltage change in the model in response to the triangular current pulse used in recordings. Current amplitude set to produce a somatic voltage change of approximately 9 mV. **(F)** Attenuation of EPSP amplitude with respect to distance of stimulation site from soma.

To investigate a possible role for high voltage-activated potassium channels, we constructed a conductance-based compartmental model based on dual somatic and dendritic patch clamp recordings (Figure 7A,B). We constrained the model with the measured cellular parameters somatic input resistance (6 MΩ) and resting membrane potential (-68 mV). Both parameters were consistent between the recordings and the model (Figure 7C,D). The modeled morphology represents a simplified version of the adult MSO dendrite (Rautenberg et al., 2009) and consisted of a single somatic compartment and two tapering dendrites (see section “Materials and Methods”). In the following, three versions of the model were tested, one with only low voltage-activated potassium channels (“KLT”), one with only the DTX insensitive potassium currents we measured (“KHT”), and one with a mix of both (“KLT + KHT”). For the mixed model, we reduced the maximum conductance of each potassium current to maintain the passive membrane properties. Both conductances were reduced by an equal percentage from the values in their respective single potassium current models. This necessitated an adjustment of the HCN peak conductance as indicated in the section “Materials and Methods.” Although both potassium conductances activate at resting potential their main distinction is the steeper activation curve of the KLT. We repeated all simulations with two different time constants for the KHT gating variable (see section “Materials and Methods”). We found no qualitative difference between the simulations using either time constant. All results reported in this section are from simulations using the faster time constant of 0.8 ms for activation and deactivation.

The passive properties varied along the dendrite to a different extent between models. The KLT model showed a greater increase in input resistance compared to the KHT model, with the mixed model intermediate between the two. Nevertheless, as these differences were greatest in the distal dendrite, all models appear consistent with measured values (Figure 7C). Resting membrane potential varied only minimally along the dendrite, consistent with the recorded values, which showed no trend (linear regression, *p* = 0.38; Figure 7D).

We compared the attenuation of membrane potential deflections between recordings and each version of the model. For both patch clamp recordings and model simulations we used a triangular current pulse injected through the dendritic electrode (Figure 7A,B). The peak current magnitude used for simulations was adjusted to give an approximately 9 mV voltage change at the soma. The change in dendritic membrane potential required to produce a 9 mV somatic deflection increased with greater distance from the soma, but did not differ noticeably between model variants (Figure 7E). Attenuation therefore did not differ between model variants (Figure 7F). Attenuation was somewhat less than the recorded values from patch clamp experiments, most likely due to the absence of dendritic branching in our model, which artificially increases dendritic diameter for a fixed cell surface area.

**FIGURE 8:**
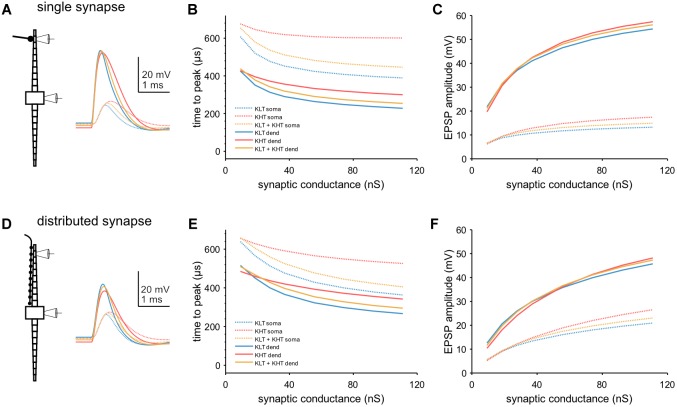
Model response to simulated synaptic stimulus. **(A)** Example model responses to EPSG of 37 nS at a single dendritic site. Traces from stimulated dendritic (solid lines) and somatic (dotted lines) compartments as indicated by pipette icons. Blue = KLT, red = KHT, and yellow = KLT + KHT. **(B)** EPSP peak times relative to EPSG onset for a single, distal dendritic input. **(C)** Amplitude of EPSPs with respect to peak synaptic conductance for a single, distal dendritic input. Legend same as **(B)**. **(D)** Same as **(A)**, but with synaptic input spread evenly across all compartments of one dendrite. **(E)** EPSP peak times relative to EPSG onset for synaptic input distributed across the full dendrite. **(F)** Amplitude of EPSPs with respect to peak synaptic conductance for distributed synaptic input. Legend same as **(E)**.

We next looked at EPSP kinetics and observed a difference in peak timing between variants of the model when we simulated single EPSGs [peak conductance = 37 nS; (Couchman et al., 2010)] at a single distal dendritic compartment (Figure 8A). We found that peak time was latest in the KHT only model and earliest in the KLT only model over a range of synaptic conductances (Figure 8B). The mixed model peak times at the soma were consistently just under 60 μs later than the KLT model over the range of synaptic conductance. Somatic EPSP amplitudes were highest for the KHT only model, which were up to 4 mV larger than in the KLT only model, although the differences were smaller for smaller synaptic strengths (Figure 8C). Amplitudes in the mixed model were generally intermediate between the other two variants.

We repeated these simulations with the synaptic conductance distributed among all compartments along the dendrite (Figure 8D). Total synaptic conductance was the same as for stimulation at a single synaptic site. With this synaptic configuration, both peak timing and amplitude showed the same qualitative trend between each model variant (Figure 8E,F). Differences in somatic peak times were somewhat closer between variants with this synaptic configuration, with 40–50 μs between the KLT only and mixed models for most synaptic strengths. In summary, KHT channels seem to trade latency for amplitude, where latency and amplitude are reduced the less KHT is in the model, due to the consequently larger amount of KLT.

**FIGURE 9:**
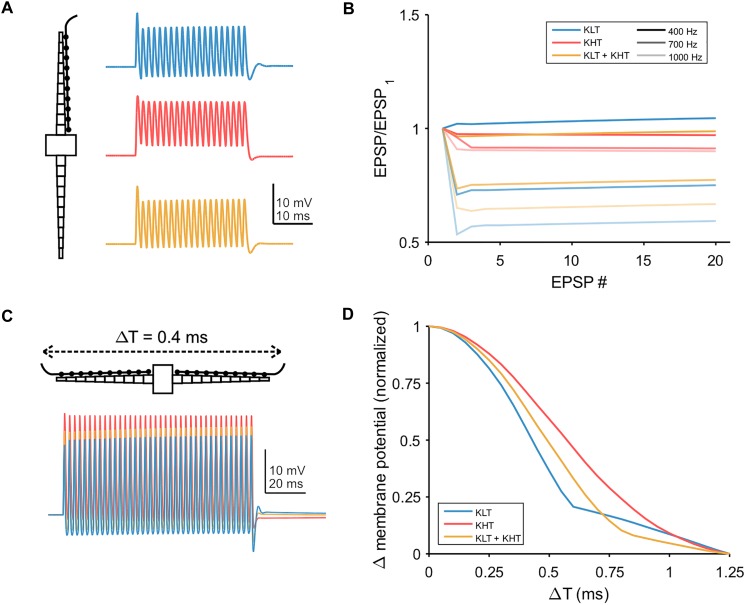
Temporal integration in model. **(A)** Example traces showing the somatic response to a train of twenty EPSPs at 700 Hz, with synaptic conductance distributed across one dendrite. Blue = KLT, red = KHT, and yellow = KLT + KHT. **(B)** EPSP amplitude divided by the amplitude of the first EPSP for each EPSP in the train at three different frequencies. **(C)** Simulations of 100 ms trains of EPSGs distributed along the length of each dendrite. Somatic voltage response shown. EPSP frequency = 400 Hz, time difference of EPSG onset between dendrites = 0.4 ms. **(D)** Peak membrane potential depolarization with respect to the difference in input timing between dendrites (Δ*T*). Measured at the end of the EPSP train. Peak membrane potential depolarizations (Δ*V*_m_) for each model version were normalized to the range 0–1 according to the following equation: Normalized Δ*V*_m_ = [Δ*V*_m_ – min(Δ*V*_m_)]/[max(Δ*V*_m_) – min(Δ*V*_m_)].

We also simulated trains of synaptic input, with the synaptic conductance distributed throughout the dendrite (Figure 9A). We first simulated a train of EPSGs (peak total conductance = 37 nS) on one dendrite and looked at how the peak membrane potential varied throughout the train (Figure 9B). For a lower input frequency (400 Hz) each model variant showed minimal change in EPSP amplitude throughout the train. For higher frequencies, models containing KLT responded with an initial larger EPSP followed by a reduction in amplitude for the remaining EPSPs, by up to about 40% for the KLT only model at 1000 Hz. The mixed model followed this trend, with somewhat smaller amplitude reductions, while the KHT only model did not.

Finally, we simulated bilateral synaptic inputs (400 Hz, 100 ms, peak total conductance per dendrite = 37 nS) and varied the relative timing of inputs between each dendrite (example in Figure 9C). We measured the peak membrane potential depolarization from rest at the end of the EPSG train and plotted the normalized response relative to the peak at zero time difference (Figure 9D). The half-width of the response function was smallest for the KLT only model (0.43 ms) and largest for the KHT only model (0.61 ms), with the mixed model again falling in between the other variants (0.51 ms). These results corroborate our previous conclusion that the benefit of KHT channels is mostly reflected by the increase in the response amplitude (Figure 9C), but reduces the temporal resolution of the coincidence curve.

## Discussion

Here we show the diversity of spatial distribution profiles of potassium channels expressed in MSO neurons of mature gerbils. MSO neurons express low and high voltage-activated potassium channel sub-units in distinct spatial patterns. Sub-units for each of these current types are present in each dendro-somatic compartment of the MSO neuron, yet in different combinations and weights. Low voltage-activated potassium channels are more biased toward the soma, whereas high voltage-activated potassium channels also exhibit strong dendritic contributions. The presence of these types of channels and their contribution to the resting input resistance was verified pharmacologically. Computational modeling based on current kinetics indicated that high voltage-activated ion channels can modulate EPSP timing and amplitude, thereby directly affecting the binaural coincidence detector mechanism.

### Low Voltage-Activated Potassium Channels

We show that, in addition to Kv1.1, the low voltage-activated potassium channel sub-units Kv1.2 and 1.6 are expressed in neurons of the mature MSO, similar to neurons of the medial nucleus of the trapezoid body (Dodson et al., 2002; Brew et al., 2003). These sub-units show different expression profiles along the dendro-somatic axis. Kv1.1 and 1.6 are predominantly localized to the soma and proximal dendrites. Conversely, Kv1.2 localization was also present at the dendrite. Overall, low voltage-activated potassium channels appeared localized more to the soma and proximal dendrite. This expression profile agrees with pharmacological data (Mathews et al., 2010). Since Kv1.x sub-units can form heteromultimers (Sheng et al., 1993), our data indicate that the low voltage-activated potassium current is most likely a heteromultimer based on at least Kv1.1, Kv1.2 and Kv1.6 sub-units, as in the medial nucleus of the trapezoid body (Dodson et al., 2002; Brew et al., 2003) and octopus cells (Cao and Oertel, 2017).

Functionally, as indicated by the application of DTX, the low voltage-activated potassium channel mediated currents decrease the input resistance, the membrane time constant, and the EPSP half width, and shorten action potential and IPSP duration in MSO neurons (Scott et al., 2005; Mathews et al., 2010; Baumann et al., 2013; Myoga et al., 2014). Besides the somatic bias, some low voltage-activated potassium channels were also located distal to the soma. Therefore, low voltage-activated potassium currents might possess additional functions at more distal dendrites where they could directly interact with distal rapid AMPA-receptor mediated synaptic excitation (Couchman et al., 2010). Moreover, our present data support the functional role of low voltage-activated potassium channels, especially Kv1.1 and Kv1.6, in reducing the time course of IPSPs (Baumann et al., 2013; Myoga et al., 2014). This support is based on the matching expression profiles of these channel sub-units with GlyT2, a marker for inhibitory synapses, suggesting local interactions during voltage signaling.

### High Voltage-Activated Potassium Channels

We demonstrate here, pharmacologically and by immunofluorescence, the expression of high voltage-activated potassium channels in mature MSO neurons. The presence of Kv2.x and Kv3.x is consistent with a TEA sensitive current and the remaining DTX, TEA and 4-AP insensitive current (Johnston et al., 2010). Such a current has also been recently documented in MSO neurons of juvenile mice (Fischl et al., 2016). In mice this current supports the repolarization of a large action potential not present in gerbil MSO neurons. Kv2.x expression is less well documented in the auditory brainstem and was only recently found to be particularly strong in the medial and ventral nucleus of the trapezoid body (Johnston et al., 2008; Tong et al., 2013). While labeling was predominant in the axonal initial segments of MNTB neurons it was found somatically in the VNTB. Here we now present dendritic labeling of Kv2.x in the MSO of adult gerbils. Our Kv3.1b stainings indicate a species difference to rats, where expression of Kv3.1 RNA was not found in the MSO (Li et al., 2001). On the other hand, our pharmacological data indicating the presence of KV3.x in adult MSO neurons of gerbils matches the reported presence of Kv3 in juvenile MSO neurons of mice (Fischl et al., 2016). In regard to the distribution profiles, Kv2.1, Kv2.2 and Kv3.1b were predominantly expressed at the dendrite and only Kv3.2 was biased strongly to the soma of adult MSO neurons. The similar distribution pattern of Kv2.1 and Kv2.2 in the MSO might indicate that these sub-units form heteromultimers (Blaine and Ribera, 1998). Furthermore, speed of the activation and deactivation kinetics recorded at the soma is more in line with Kv3.x than Kv2.x channels. It can be speculated that the rapid kinetics at the soma are due to the differential distribution patterns of the distinct high voltage-activated potassium channels.

The expression of high voltage-activated potassium channels is unexpected in neurons that are well known to have only very little action potential backpropagation and small action potential amplitudes *in vitro* (Scott et al., 2005, 2007; Chirila et al., 2007; Lehnert et al., 2014). Yet our electrophysiological data suggest that these channels contribute to the resting input resistance, consistent with activation at low voltages. As our data is based on insensitive DTX currents our interpretation might be slightly contaminated if DTX insensitive low voltage-activated potassium channel subunits such as Kv1.3, Kv1.4 or Kv1.5 were present in MSO neurons. Now with the assumption that DTX blocks all low voltage-activated potassium channels in MSO neurons, our computational model indicates that, even at resting levels, high voltage-activated potassium channels contribute to setting the membrane potential, as they were in competition with a low amount of low voltage-gated potassium channels. Nevertheless, due to their steeper activation curve, low voltage-activated potassium channels dominated the dynamics of the mixed KLT + KHT model membrane potential.

The addition of KHT, and concomitant reduction of KLT, appears to slightly “worsen” the performance of our model neuron in measures commonly considered important for MSO neurons (Scott et al., 2005; Mathews et al., 2010). KHT induces a peak delay of up to 60 μs, which is a relevant time delay for ITD processing (Lesica et al., 2010). It is not clear, however, whether this additional latency is detrimental for ITD processing. Indeed, *in vitro* recordings using conductance clamp suggest that slower EPSGs can allow the cell to more effectively modulate the timing of peak membrane depolarization (Myoga et al., 2014). Given that MSO neurons operate at an extreme in terms of the magnitude of membrane conductances and the brevity of time constants, KHT may help counterbalance the effects of the large DTX-sensitive KLT currents, and modulate the neuronal excitability to bilateral inputs.

Our study shows that voltage signaling in MSO neurons is based on many different potassium channels with distinct spatial expression profiles in the somato-dendritic compartments. As the focus of this study was on the somato-dendritic compartments, which carry out the voltage signaling underlying ultrafast coincidence detection, their expression and contribution to the voltage signaling in the axonal compartment and therefore to action potential generation remains unanswered. In spite of the lack of overshooting action potentials in the somato-dendritic compartments of these neurons, they still possess a full set of voltage-activated potassium channels whose full function we have just started to disentangle.

## Author Contributions

AN and SAG performed immunofluorescence. AC, NK, and FF performed electrophysiology. AC and CL performed computational modeling. AN, AC, NK, FF, and CL analyzed and interpreted data. SAG, CL, and FF devised experiments, AN, AC, CL, and FF wrote the manuscript.

## Conflict of Interest Statement

The authors declare that the research was conducted in the absence of any commercial or financial relationships that could be construed as a potential conflict of interest. The reviewer JS and the handling Editor declared their shared affiliation
